# Study of Organosolv Lignins as Adhesives in Wood Panel Production

**DOI:** 10.3390/polym9020046

**Published:** 2017-01-29

**Authors:** Georges Koumba-Yoya, Tatjana Stevanovic

**Affiliations:** Centre de recherche de sur les Matériaux renouvelables, Département des Sciences du bois et de la forêt, Université Laval, Québec, QC 7337, Canada; georges-thibaut.komba-yoya.1@ulaval.ca

**Keywords:** organosolv lignin, sugar maple bark and wood, adhesive performance

## Abstract

Organosolv lignins obtained from sugar maple bark and wood were studied as adhesives for wood particleboard production. Organosolv pulping of sugar maple wood and bark was carried out in the presence of Lewis acid FeCl_3_ as a catalyst. The organosolv lignins recovered from this process were investigated by determination of Klason plus acid-soluble lignin content, of sugars by HPLC analysis, and of ash content. Structural characterizations of these lignins were performed by Fourier-transform infrared (FT-IR) and by ^31^P NMR. The results of the latter studies indicate that the content of free phenolic groups was more important in bark than in wood lignin. The gel permeation chromatography (GPC) analyses results suggested that the weight-average molecular mass of wood lignin was higher than that of bark lignin. The studied organosolv lignins were used for the preparation of particleboards as recovered and in combination with glyoxal or isocyanate. It was found that sugar maple bark lignin, as such or modified with isocyanate, was a more efficient adhesive than its wood counterpart. On the contrary, it was the organosolv wood lignin combined with glyoxal which was a more efficient adhesive than its bark counterpart. In combination with isocyanate, it was the sugar maple bark organosolv lignin which was determined to have the best adhesive performance of all studied lignins.

## 1. Introduction

The renewed interest in developments related to green chemistry and promotion of products from biomass is due to two main factors: depletion of petroleum feedstocks and need for sustainable development, implicating reduced emissions of greenhouse gases. It is in this context that research on forest biomass, based on principles of sustainable chemistry, is leading to perpetual changes towards innovative products. In this context, promoting complete utilization of wood biomass, from extractives to structural biopolymers, represents an important strategy of competitiveness in the context of sustainable development, bringing economic benefits to forest industry. Considered as waste issued from wood transformation, bark has received little attention in the past. Thus, in order to promote the valorization of wood and bark, several studies have revealed the adhesive properties of lignin [1]. On the other hand, due to the low purity and quality of lignin, industrial uses of lignin as adhesive resin are still at the research and development level. Indeed, lignin recovery is difficult to control efficiently because, so far, extraction processes implemented to isolate lignin usually end up destroying its primary (native) structure. In fact, to understand the molecular structure of lignin, models of lignin as dimers of β-O-4 type have been synthesized and commonly used to study various lignin reactions. Generally, lignin extraction from lignocellulosic materials is carried out under conditions in which lignin is gradually fragmented into lower molar mass products, causing the change of its physicochemical properties in comparison to those of its native state. Currently, the industrial lignins are available from black liquors from sulfite and kraft pulping. Despite a high potential production capacity of kraft and sulfite processes, the high levels of carbohydrates, ash, and sulfur content in lignin issued from these processes seriously limit their applications. On the other hand, the organosolv lignin, with low carbohydrate content, have been produced on a larger scale for only a limited time in the past, and are currently not produced on a large scale [2,3]. However, the interest for organosolv processes remains important, mostly because these processes usually provide lignins of higher purity, suitable for multiple potential applications. Organosolv lignin extraction process consists of solubilization and extraction of lignin and hemicelluloses with an organic solvent (typically methanol or ethanol), leaving behind insoluble solid cellulose fibers [4,5,6]. The use of renewable resources in the materials industry is increasingly investigated not only to allow for reduction of energy consumption, but also to decrease emissions of the toxic substances from the final product. Thus, wood panel production is under pressure to reduce production costs and harmful emissions related to resins derived from the petroleum industry. Numerous works reported the production of lignin-based resins for wood panels. Lignins contain several functional groups such as hydroxyl, both phenolic and aliphatic OH, which have similar properties as petrol-derived phenol. Thus, lignin was considered as the most promising substitute of phenol-targeting adhesive applications [7]. However, lignin is an amorphous polymer which has low reactivity towards crosslinking reactions. The isolated lignins are generally poor binders for wood panels when compared to common resins such as phenol formaldehyde (PF) resins [8]. Several lignin activation reactions have been explored to enhance its reactivity as adhesive resin, among which are a pre-reaction by demethylation [9] or activation with glyoxal [10] or with isocyanate [11]. In this work, we have applied a new organosolv process with an ethanol–water system, using FeCl_3_ as a catalyst, on sugar maple wood and bark [12]. The adhesive properties of thus obtained organosolv lignins were studied—applied solely or in combination with glyoxal and isocyanate—in particleboard preparation.

## 2. Materials and Methods 

### 2.1. Wood Grinding and Chemical Composition Determination

Sugar maple (*Acer saccharum*) bark and wood were provided by Decacer Inc. Wood and bark were ground to particles sized between 40 and 60 mesh prior to chemical analyses. After drying at 80 °C for 24 h, these particles were used for organosolv pulping experiments. Ferric chloride, sulfuric acid (95%–97%), and 4,4′-methylenebis(phenyl isocyanate) (98%) were used as purchased from Sigma Aldrich. Glyoxal (40% in water) was purchased from Laboratoire Mat Inc. Ethanol (95%) was purchased from Commerce Inc. and hexamethylenetetramine (HMTA, 99%) was purchased from Sigma Aldrich. Trembling aspen wood particles of 60 μm were chosen to produce the composite panels. Extractives content was determined according to ASTM method, using ethanol–toluene (ASTM D1107-96) for both bark and wood, followed by hot water-soluble (ASTM D1110 84) extractives determination applied for wood and by 1% NaOH extraction for bark (ASTM D1109-84). After these treatments, biomass compositions were evaluated by lignin quantification (Klason and acid-soluble lignin) according to standard methodology ASTM D1106 and carbohydrates analyses performed according to National Renewable Energy Laboratory (NREL) methodology (Determination of Structural Carbohydrates and Lignin in Biomass). 

### 2.2. Moisture and Ash Content of Samples

The moisture content of samples was determined by weight loss after oven-drying at 105 °C following the standard methodology (ASTM 1102-84). For each sample, this analysis was repeated in triplicate. The ash content was determined using ventilated muffle oven under a controlled temperature program from room temperature to 600 °C for 8 h. 

### 2.3. Extraction Prior to Organosolv Pulping

Prior to delignification, the wood particles were pretreated with an ethanol–water mixture (1:1, *v*/*v*; 1 L of final volume mixture for 100 g of bark or wood) at 80 °C for 6 h in a Soxhlet extractor, in order to remove phenolic and other extractive compounds along with volatile materials, which could damage lignin purity. After filtration, extractive-free wood particles were dried at 80 °C for 24 h prior to pulping. 

### 2.4. Delignification of Extractives-Free Biomass 

The extractives-free wood and bark were treated under the same pulping conditions, using ethanol–water mixture (1:1, *v*/*v*; 0.5 L of final volume for 50 g of wood) in Parr reactor series 4842 (2 L), using ferric chloride as a catalyst at 180 °C for 90 min (6 mmol of FeCl_3_·6H_2_O for wood and 9 mmol for bark). After cooking, the reaction mixture was left to cool down to room temperature. The obtained organosolv pulp was separated by filtration and washed three times with ethanol (200 mL) to recover all dissolved lignin. The organosolv lignin was precipitated from dark-brown liquor by acidification with 2 M HCl to pH = 1.5. The precipitated organosolv lignin was filtered and dried under vacuum at 40 °C overnight. Organosolv lignin was obtained in form of solid brown powder.

### 2.5. Determination of Lignin and Carbohydrates in Wood and Bark 

Lignin quantification (Klason and acid-soluble lignin) was performed according to standard methodology ASTM D1106. Carbohydrate analyses of wood and bark samples were carried out in triplicate following the NREL methodology for monosaccharides quantification by HPLC-RID (refractive index detector) using an Agilent Technologies 1200 series equipped with a Rezex RHM-Monosaccharide H+ (8%) (300 × 7.8 mm) column. Elution with deionized water at 0.5 mL/min was performed for 20 min at 75 °C. The standard calibration curve was obtained using the standards of cellobiose, glucose, xylose, mannose, and arabinose (Sigma-Aldrich). The identification and quantification of sugars were performed by retention time (tr) with injection, at four points, using chromatographic grade standards at different concentrations. 

### 2.6. Gel Permeation Chromatography Analyses of Organosolv Lignins

The molecular weights distributions of the organosolv lignins were determined using solutions of 20 mg of lignin in 2 mL of tetrahydrofuran (THF), which were filtered through 0.45 μm porosity filter prior to injection. The gel permeation chromatography (GPC) analyses were performed at 50 °C on an Agilent 1200 series equipped with column PLgel 5 μm Mixed-D 300 × 7.5 mm and using THF as eluent (0.5 mL/min). A diode-array detector was used at 254 nm. The molecular masses are presented relative to polystyrene standards (from 580 to 28,770 Da, Agilent).

### 2.7. FT-IR Analyses

Normalized Fourier-transform infrared (FT-IR) spectra were obtained for each lignin sample using an FT-IR spectrometer attenuated total reflectance (ATR)-FT-IR/FT-NIR (near infrared) PerkinElmer Spectrum 400). The FT-IR spectra were recovered for 64 scans and collected for wavenumbers ranging from 4000 to 650 cm^−1^.

### 2.8. NMR Analyses of Lignins 

HSQC NMR spectra were recorded on a Varian NMR spectrometer at 500 MHz on solution obtained by dissolving 80 mg of lignin in 0.5 mL of dimethylsulfoxide (DMSO)-D6. Data processing was performed using MestReNova 8.1.1 software. Quantitative ^31^P NMR using published procedures [12] were used. The ^31^P NMR spectra were recorded on a Varian NMR spectrometer at 500 MHz at 256 scans by dissolving 40–45 mg of oven-dried (OD) lignin in 0.5 mL of anhydrous pyridine/CDCl_3_ mixture (1.6/1, *v*/*v*). A total of 0.1 mg of an endo-*N*-hydroxy-5-norbornene-2,3-dicarboximide for each milligram of lignin was added as the internal standard, and 0.06 mg of chromium (III) acetylacetonate for each milligram of lignin as the relaxation reagent. Finally, 150 μL of 2-chloro-4,4,5,5-tetramethyl-1,2,3-dioxaphospholane was added as phosphitylating reagent and transferred into a 5 mm NMR tube for NMR analysis.

### 2.9. Thermogravimetric Analyses (TGA)

Thermogravimetric analyses of lignin samples were performed on TGA/SDTA851 from Mettler Toledo following the procedure previously described [12]. Thermogravimetric analyses were conducted first under air from 25 to 250 °C, followed by carbonization at 25–800 °C under nitrogen. The heating under air to 250 °C was performed at a rate of 5 °C/min, and the sample was then maintained at 250 °C for 30 min to allow for the stabilization and the oxidation of lignin. The sample was then cooled to 25 °C and then heated to 800 °C at a rate of 5 °C/min under nitrogen in order to allow for the carbonization of the sample. It was finally maintained at 800 °C for 30 min.

### 2.10. Lignin-Based Resin Preparation

The glyoxalated lignin was prepared according to published procedure with slight modifications [10]. Briefly, 15 g of lignin was added to 30 g of water. Aqueous solution of sodium hydroxide (2 M) was added to obtain pH of 12. Four grams of glyoxal (10 g of glyoxal at 40% in water) was added and the mixture was stirred and heated to 70 °C for 8 h. The lignin–isocyanate [11] was prepared by combining lignin (15 g) in tetrahydrofuran (50 mL) and isocyanate (0.5 mmol of isocyanate for each gram of lignin). The mixture was kept stirring at 100 °C for 60 min. For lignin without modification and glyoxalated lignin, hexamethylenetetramine (HMTA) was prepared at 50 g/L in water and added (5 mL of HMTA at 50 g/mL in 100 g of particleboards) as a hardener.

### 2.11. Particleboard Preparation and Testing

Each particleboard sample was prepared using 15% of total adhesive resin on dry wood. Particleboards of 110 mm × 110 mm × 4 mm were prepared using trembling aspen particles which were pressed at 4 MPa and 195 °C during 7 min. Flexural tests were performed on an Intron model 5565 with a 500 N load cell at room temperature with a support span of 60 mm and a crosshead speed of 2 mm/min. All tests were performed in triplicate according to ASTM D790. 

## 3. Results

Lignin was extracted from wood and bark of sugar maple using our patented organosolv process with ferric chloride as a catalyst [12,13]. Before biomass pulping, chemical composition of wood and bark was determined applying standard protocols of wood chemistry (Table 1).

For extractive quantification from bark, ethanol–toluene extraction (ASTM) followed by hot water extraction with 1% of sodium hydroxide solution (ASTM D1109-84) was applied. For extractive quantification from wood, ethanol–toluene extraction (ASTM) followed by hot water extraction only was applied. In bark of sugar maple, extractive content with ethanol–toluene was 4.9% and 21.8% with hot water extraction with sodium hydroxide solution at 1% (26.7% of total extractives). Previous work on bark of sugar maple by Huang et al. [14] reported extractive content at 20.5% with 1% of sodium hydroxide solution. For wood extractives, ethanol–toluene extraction gave 2.1% and 3.4% for hot water extraction. A previous report [15] found 3.2% of extractive content in wood of sugar maple with hot water extraction. We determined somewhat higher Klason lignin in bark than in wood: 29.6% against 25.7%, respectively (see Table 1). This result is similar to previously reported higher lignin content in bark than in stem wood found for another hardwood, *Salix caprea* [16]. On the other hand, total carbohydrate content (glucan and xylan) was determined in this study to be higher in wood than in bark. These results were in accordance with previous works [16]. Finally, bark was determined to have higher ash content than sugar maple wood studied here.

The organosolv lignins were extracted from bark and wood using the organosolv process as described in our previous work [12], using an aqueous ethanol solution (50%) and ferric chloride as a catalyst. In this study, catalyst at 6 mmol was applied for wood pulping and 9 mmol for bark. After pulping at 180 °C for 90 min, 24.1% and 21.2% yields of lignin from bark and wood, respectively, were determined. The lignin recovery was based on Klason lignin of original bark (81.4%) and was slightly lower than the lignin recovery from wood (82.5%). The results of organosolv lignin purity determination are presented in Table 2.

All lignins present a high Klason lignin above 90%. According to sugar and ash content, the bark lignin purity is higher than that of wood lignin. On the other hand, the higher level of acid-soluble lignin content in bark lignin indicates that the organosolv conditions contributed to more important cleavage of aryl–ether bonds than in wood lignin. Besides lignin purity, the high content of sugar in wood lignin confirms that the high level of catalyst can promote extensive cleavage of carbohydrate hydrolysis, as previously reported, causing their subsequent deposit on lignin [12].

**Figure 1:**
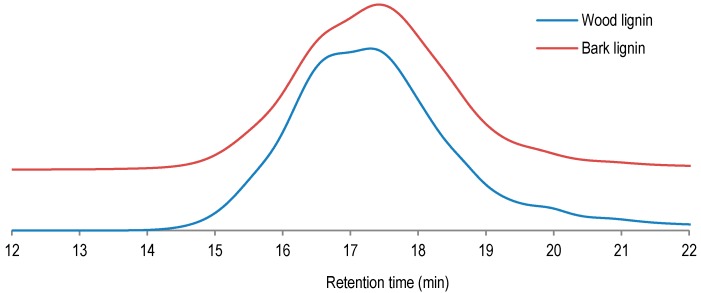
Comparative gel permeation chromatography (GPC) analysis of wood and bark lignin.

As shown in Figure 1 and Table 3, the studied lignin samples are determined to have similar average molecular weight distributions of 1755 g/mol for wood lignin and 1654 g/mol for bark lignin. The lowest polydispersity (PD) was determined for bark lignin (2.73 against 3.47 of PD for wood lignin). The lower average molecular weight and lower polydispersity of bark lignin could be an indication of more pronounced prepolymer properties of bark lignin compared to wood lignin. These prepolymer properties could be exploited for subsequent polycondensation and polymerization during curing and panel pressing. Indeed, in phenol adhesive application, Prepolymers are determined to have higher reactivity and to be more susceptible to crosslinking and copolymerization [17].

**Figure 2:**
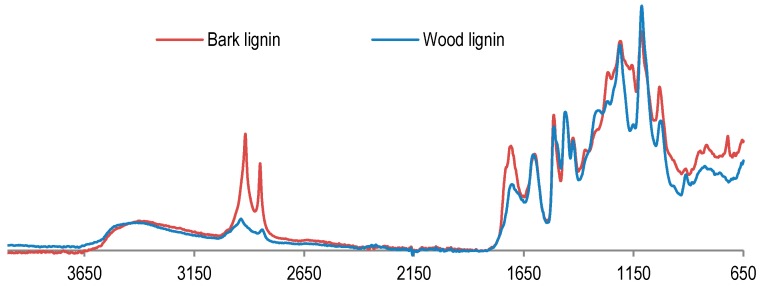
Fourier-transform infrared (FT-IR) analyses of organosolv lignin from wood and bark.

**Figure 3:**
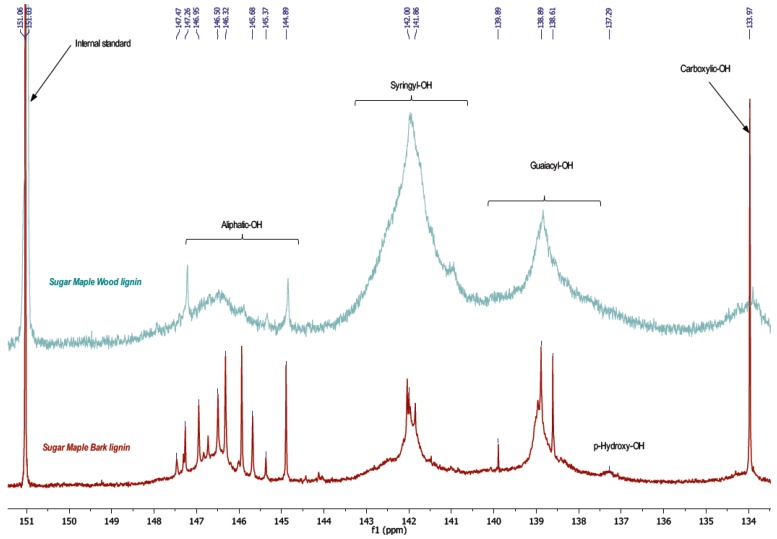
Comparative ^31^P NMR spectra of original lignin from bark and wood.

In order to examine lignin properties, a comparative study between wood and bark lignin was performed by FT-IR analyses (Figure 2). FT-IR spectra present the difference between wood and bark lignin. The signal at 2919 cm^−1^ is assigned to OH from aliphatic or carboxylic acid is more prominent in the bark lignin spectrum. The same is found for the signal at 1717 cm^−1^, attributed to absorption of carbonyl groups from ester, ketone, aldehyde, or carboxylic acid. These results indicate that bark lignin could contain more aliphatic hydroxyl and carbonyl groups from carboxylic acid. This hypothesis has been confirmed by the results of phosphorus NMR analysis of lignin samples presented in Figure 3. 

The results by phosphorous NMR study reveal two principle pieces of information concerning phenol and aliphatic hydroxyl content. Indeed, an important difference between wood and bark lignin is revealed in the aliphatic hydroxyl region, between 144.5 and 148 ppm, and an intense peak of carboxylic hydroxyl, around 134 ppm, which are higher in bark than in the wood lignin spectrum (Figure 3, Table 4). These results confirm the FT-IR assignments. 

Except for the aliphatic hydroxyl in the bark lignin spectrum, the detected signals displayed the fragments such as phenylcoumaran units formed by β-5 substructures typical for lignin polymer in progress of growth. The lower molecular weight of bark lignin confirms this result. The peak around 139 ppm is attributed to the guaiacyl unit with free phenolic hydroxyl, while the peak at 142 ppm corresponds to phenolic hydroxyl present in syringyl units. In bark lignin, syringyl and guaiacyl units were determined in similar quantity, while these same signals were very different in wood lignin. In wood lignin, syringyl units might be associated higher condensation structures such as β-β, β-5, and 5-5. Indeed, these condensed structures appear in the same region between 141 and 143 ppm [18].

**Figure 4:**
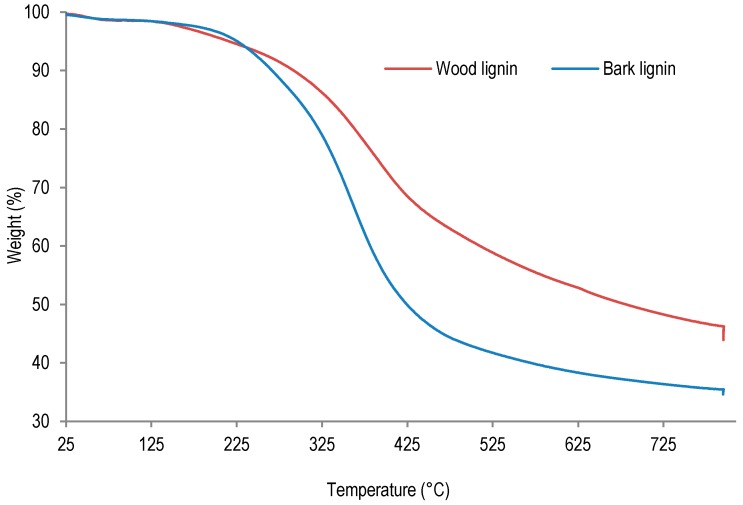
Thermogravimetric analysis (TGA) analysis of lignins samples from sugar maple.

In order to examine the thermal behavior of lignins from wood and bark, these samples were studied by thermogravimetric analysis (TGA). The TGA analyses results are presented in Figure 4 and Table 5.

The thermogravimetric analysis of lignin samples was performed between 25 and 800 °C under nitrogen flow. As the results presented in Table 5 indicate, the temperature of first thermal degradation of wood lignin is much lower (146 °C) than corresponding temperatures determined for bark lignin (208 °C). This result suggests an extensive depolymerization of wood lignin or residual sugar degradation during this thermal ramp. Indeed, according to lignin purity analysis, wood lignin contained more residual sugar than bark lignin (see Table 2). On the other hand, the temperature corresponding to 50% weight loss was determined to be 410 °C for bark lignin, whereas the temperatures corresponding to thermal degradations leading to 50% weight loss of wood lignin was determined to be 653 °C. These results suggest that after depolymerization during first degradation at 145 °C, repolymerization can take place in wood lignin, leading to a higher thermal stability at 653 °C. Beside lignin repolymerization, the higher ash content (0.80% against 0.08%, Table 2) could also contribute to higher thermal stability of wood lignin.

**Figure 5:**
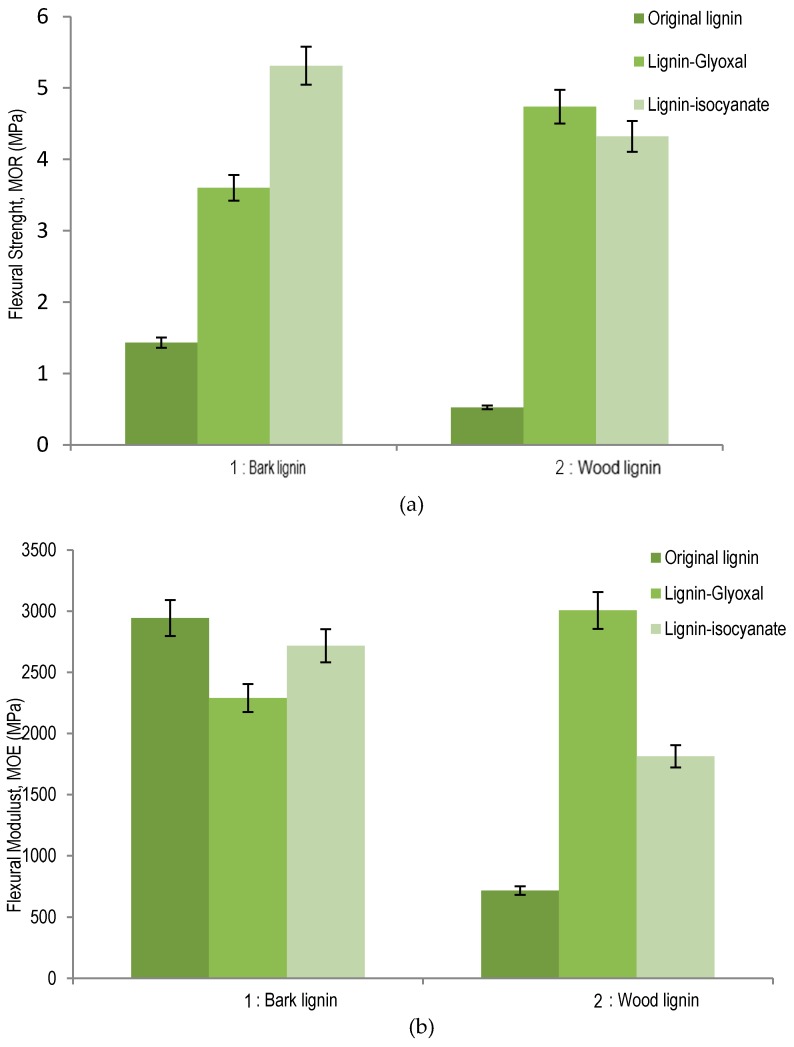
Influence of lignin and modified lignin in particleboards panel. (**a**) Modulus of rupture (MOR) values of particleboard panels; (**b**) modulus of elasticity (MOE) values of particleboard panels.

The adhesive performance of studied wood and bark organosolv lignins, as such and modified with glyoxal or isocyanate, was tested by adding 15% of each lignin sample in particleboards curing at 195 °C for 7 min of press time. The results of comparison between bark and wood organosolv lignins are summarized in Figure 5 for modulus of rupture (MOR) strength and modulus of elasticity (MOE). The Figure 5a shows the effect of lignin as resins on the MOR measurement of panels. Particleboard panels pressed with organosolv lignin from sugar maple bark (1.43 MPa) exhibited better bending strength than original wood lignin (0.52 MPa). When lignin was modified with glyoxal, particleboard bonded with sugar maple wood lignin–glyoxal (4.74 MPa) was determined to have better MOR strength values than the bark lignin–glyoxal (3.60 MPa) panel counterpart. Wood lignin–glyoxal exhibited a significant increase of MOR compared to original lignin. Thus, glyoxal showed more affinity for sugar maple wood lignin than for its bark lignin. Indeed, the evidence that the lignin substitution occurred at C_5_ position in the aromatic ring can explain the increase of MOR strength in particleboards. For lignin–isocyanate, the best results were obtained with bark lignin (5.31 MPa) comparative with lignin–isocyanate from wood (4.32 MPa). These results could be associated with the high content of aliphatic OH of bark lignin, which reacts as polyol with isocyanate for polyurethane linkage formation. The mechanisms of lignin–glyoxal and lignin–isocyanate are presented in the discussion section.

The measured MOE of particleboard panels with different lignin samples are summarized in Figure 5b. Surprisingly high MOE was observed for wood panels prepared with bark lignin only (2943 MPa), which was determined to be higher than that determined for panel prepared with bark lignin–glyoxal combination (2290 MPa) or bark lignin–isocyanate (2717 MPa). The comparison of MOE levels between panels prepared with unmodified organosolv lignins indicates that sugar maple bark lignin (2943 MPa) performed much better as adhesive than wood lignin (718 MPa). These results indicate that high content of condensed units determined in wood lignin could have contributed to the decrease of elasticity and increase of the rigidity of particleboards prepared with sugar maple wood organosolv lignin. For lignin–glyoxal combination, the best results of MOE were obtained for wood lignin (3005 MPa). Compared to wood lignin–glyoxal, the lignin–isocyanate panel was determined to have a significantly lower MOE (i.e., elasticity performances (1813 MPa)), while an addition of isocyanate to bark lignin contributed to a panel with a much better performance, with a MOE at 2717 MPa. 

## 4. Discussion

**Figure 6:**
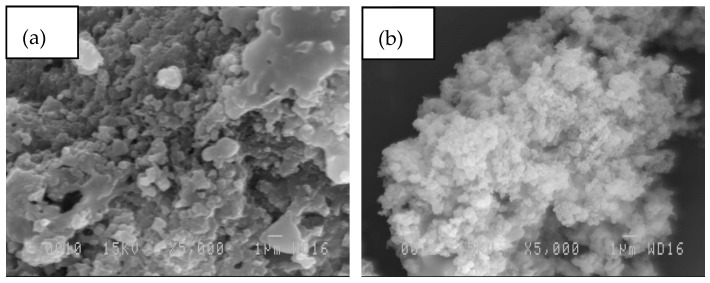
SEM analyses of recovered lignin. (**a**) Bark lignin; (**b**) wood lignin.

Lignin recovery (81.4% from bark lignin and 82.5% from wood), indicates that the organosolv lignins were successfully extracted from sugar maple in presence of ferric chloride as a catalyst. Lignin purity (Klason above 90%), was determined to be satisfactory, indicating a low residual sugar content, consisting principally of glucose (see Table 2). The examination of recovered organosolv lignin was carried out also by SEM analyses (Figure 6).

**Figure 7:**
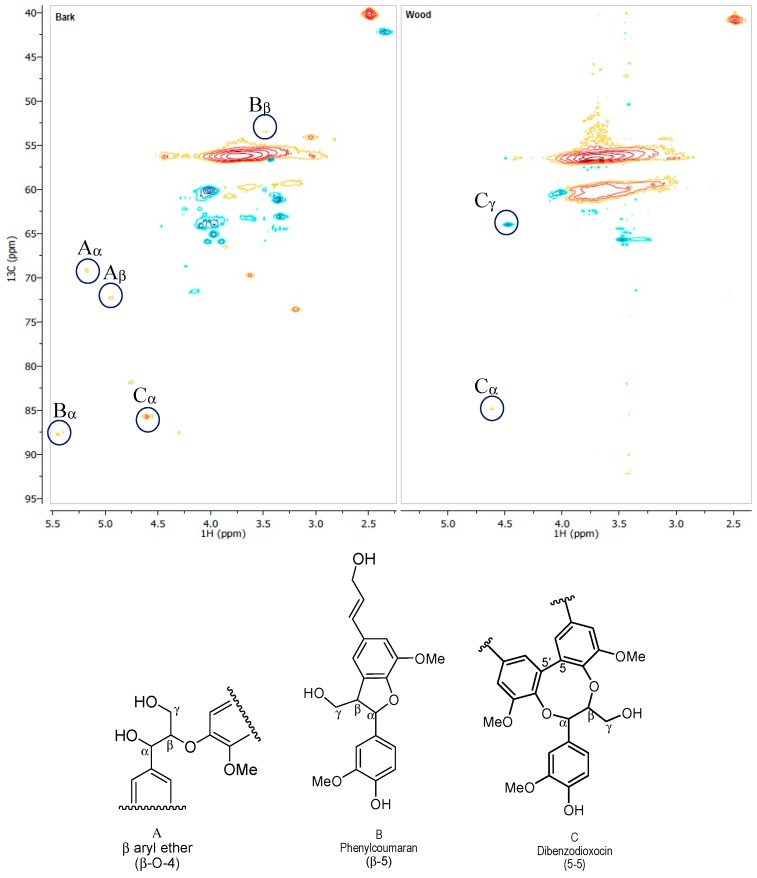
Comparative HSQC analyses of original lignin from bark and wood.

Bark lignin showed more bulk particles (Figure 6a), while wood lignin presented finer particles, which appeared to be highly condensed. Comparative ^31^P NMR experiments revealed a high condensed aromatic content associated to syringyl units in wood lignin. It was observed that bark lignin exhibited the lower condensed structure with high phenylcoumaran moiety content. The lower intensity of the peak between 144.5 and 148 ppm in wood lignin might illustrate a condensed dibenzodioxocin-type structure that has been reported to be concentrated in low molecular mass lignin fragments (Figure 7) [19]. Indeed, HSQC analysis has shown a signal which corresponded to dibenzodioxocin as a major fragment in wood lignin.

The major compound in wood lignin revealed a much-condensed unit such as dibenzodioxocin, which was more difficult to solubilize in deuterium solvent for NMR experiments. In accordance to the high molecular weight of wood lignin determined by GPC, a successive depolymerization/repolymerization of this lignin is hypothesized to take place due to the use of ferric chloride at high concentration, which could have catalyzed crosslinking reactions of sugar maple wood lignin.

**Figure 8:**
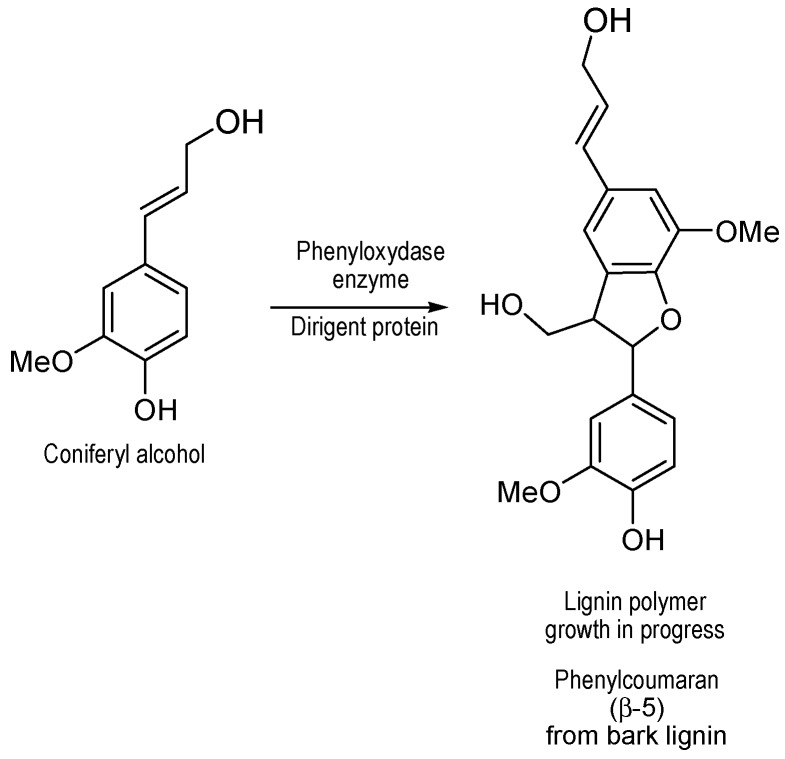
Proposed structure of major fragment in bark lignin. Adapted from [20].

On the other hand, ^31^P NMR experiments (Figure 3), which revealed the high level of aliphatic hydroxyls in bark lignin as also identified in HSQC (Figure 7), could be explained by the growth of lignin polymer from coniferyl alcohol coupling in presence of dirigent proteins as catalysts to produce phenylcoumaran type of structures, as previously proposed (Figure 8) [20].

Compared to our previous work [12], the high level of catalyst applied in this research could have induced important modification to the lignin structure compared to its native state. In this case, the cleavage of β-O-4 linkages was the major mechanism of lignin breakdown during pulping. 

**Figure 9:**
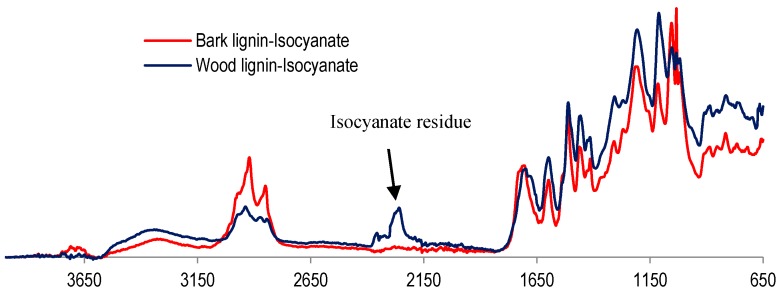
Comparative FT-IR spectra of lignin–isocyanate.

With glyoxal activation, sugar maple wood organosolv lignin was shown to have a better performance in panel application than bark lignin. Conversely, lignin reaction with isocyanate was more successful with bark lignin than with wood lignin, as determined by particleboard performance. This favorable interaction between bark lignin and isocyanate was also confirmed by the FT-IR analysis in comparison to wood lignin–isocyanate FT-IR spectrum (Figure 9).

Compared to wood lignin, bark lignin revealed no cyanate moiety, which appears at 2280 cm^−1^, confirming its reaction and the consumption of isocyanate by bark lignin (Figure 9). On the other hand, the assigned signal of the OH hydroxyl group, around 3300 cm^−1^, decreased in bark lignin after its combination with isocyanate. This could be explained by the reaction of isocyanate and bark lignin, which was completed due to the high aliphatic hydroxyl content of bark lignin, while with wood lignin this reaction was not completed.

## 5. Conclusions 

The experimental data obtained in this research demonstrated the significant difference between sugar maple organosolv bark and wood lignin. Direct glyoxalation in lignin under alkaline conditions was more efficient for enhancing the mechanical properties of wood panel when sugar maple organosolv wood lignin was used as resin. On the other hand, the results of the FT-IR analyses seem to indicate that the reaction with isocyanate was more advanced with bark lignin than with wood organosolv lignin. This could confirm the results of the GPC analyses, which demonstrated that bark lignin had lower average molar mass and, consequently, more reactive sites available for reactions taking place during panel curing. The good performance of bark lignin with isocyanate could indicate its applicability as polyols in polyurethane resin formulation for panel production. 

## Figures and Tables

**Table 1:** Chemical composition of sugar maple bark and wood (% of OD mass).

Entry	Main constituents	Bark	Wood
1	Ethanol–Toluene	4.9	2.1
2	Hot water	21.8 *	3.4
3	Extractives	26.7	5.5
4	Klason lignin	27.1	22.2
5	Acid-soluble lignin	2.5	3.5
6	Total lignin	29.6	25.7
7	Glucan	23.7	48.9
8	Xylan	15.9	19.4
9	Ash	5.2	0.40

* Hot water extraction containing 1% of sodium hydroxide solution at 50%.

**Table 2:** Comparison of sugar maple bark and wood organosolv lignins purity (composition in % of OD mass).

Entry	Main constituents	Bark lignin	Wood lignin
1	Klason lignin	94.4	95.8
2	Acid soluble lignin	3.5	2.5
3	Total lignin	97.9	98.3
4	Glucose	1.5	2.2
5	Xylose	N.D.	N.D.
6	Ash	0.08	0.80

N.D.: not detected.

**Table 3:** Gel permeation analysis for lignin samples from wood and bark.

Entry	GPC	Wood lignin	Bark lignin
1	Mw (g/mol)	1755	1654
2	Mn (g/mol)	574	615
3	PD	3.47	2.73

**Table 4:** Quantitative ^31^P NMR analysis of different lignin (OH mmol per g of lignin sample).

Entry	Functional group	Wood lignin	Bark lignin
1	Aliphatic	0.15	1.37
2	Syringyl	2.12	1.59
3	Guaiacyl	1.01	1.61
4	*p*-Hydroxyl	N.D.	0.09
5	Carboxylic	0.01	0.34

**Table 5:** TGA results performed on lignin samples under nitrogen.

Entry	Analysis	Wood lignin	Bark lignin
1	Temperature of first degradation (°C)	146	208
2	Temperature of 50% weight loss (°C)	653	410
